# Notes and Comments

**Published:** 1924-10

**Authors:** 


### NOTES AND COMMENTS.

### THE FINAL DISTRIBUTION.

AN informed optimism is the outstanding note of the second Interim Report of the Voluntary Hospitals Commission. The primary object of the report is to explain the manner in which the distribution of the Government Grant has been completed, but it also contains much that is distinctly encouraging upon the outlook. The first distribution amounted to more than £426,000, which left something over £73,000 in hand. Of this sum all but £16,866 was used to assist hospitals with serious accumulated deficits, and in the end it was decided to allocate that residuum to those which were in acute financial difficulties, but for various reasons had been unable to receive adequate assistance from the Government grant. This final distribution was helped by the Treasury giving the Commission discretion to waive the " new money " condition in exceptionally hard cases which could not otherwise have been assisted. Now that the money is spent the Commissioners are well justified in declaring that it has " produced results of great value." The grants have in many cases prevented the closing of beds, and in others have led to their reopening. In some cases, indeed, it was made a condition that they should be reopened. This is very much to the good, since there is no more melancholy sight in a hospital than a bed for which there is no money to maintain. The Commission point out that not only have the apprehensions that Government help would reduce the flow of voluntary contributions been entirely falsified, but that there have been very few cases in which hospitals have failed to raise the new money required to earn a grant. There is now, it is added, substantial ground for the hope that the voluntary hospitals will, for the most part, be able to balance their budgets—in general the deficits are decreasing. The crisis caused by the War is over, and we have now to see to it that the position is so strengthened that no new crisis shall be possible.

### MUSIC AS MEDICINE.

MUSIC, we know, hath charms to soothe the savage breast. Does it also minister to a mind diseased ? Sir Robert Armstrong-Jones declares that it does, and that those whose vocation is the care of the mind owe much to the curative agency of the emotions aroused by music. " By its means hope and courage may be raised, despondency dissipated, and healthy mental action may be stimulated." It is, indeed, the general experience that nothing more certainly touches the chords of memory than the rhythmic succession of sounds which we call music, and it is not surprising that Sir Robert should more than once have witnessed the dawn of mental restoration from an emotional interest in music. To him, however, enters Dr. Claye Shaw, who has been " much disappointed " with the results of music in the treatment of the insane. His experience suggests that dancing is a more useful curative agent because it has more effect upon the circulation. Patients who listened with apathy to classical music would dance alone if they could not get partners the moment a polka or a waltz was struck up. One does not need to be insane to listen with apathy to classical music—that kind of indifference is a very common disease—and Dr. Shaw's evidence is, after all, a point in favour of music in the asylum, so long as it is the right type of music for the purpose. We cannot hope that most, or even many, of the insane will dance themselves into sanity ; but we cannot afford to neglect the help either of music or of corybantics in the treatment of mental disease. There are necessarily many varieties of experience among alienists, and the exchange of those experiences is one of the most practical methods of extending our knowledge.

### FACTS AND FICTIONS ABOUT OPIUM.

MRS. WASHBURNE WRIGHT, the Assessor to the Opium Advisory Committee of the League of Nations, has been making some unwary statements upon the cultivation of the poppy, which have brought upon her a flood of corrections. She wants to know when Great Britain and other Powers holding possessions in the Far East, together with Japan and Siam, intend to complete the " progressive suppression " of the growth of this great drug-producing plant. She says that " China was given ten years in which to eradicate the poppy from her soil, and did it in seven." It is difficult to imagine a more grotesque statement. China is still growing nine-tenths of the total world's production of opium, and is likely to continue to grow it. Those who know something of that country and of the Chinese character never expected suppression in seven, or ten, or seventy years. Her main object was to get rid of the competition of Indian opium that the field might be clear for her own product. That having been more or less accomplished, any colourable attempt that may have been made at reduction in the beginning was long ago abandoned, and the existing civil commotions in China are highly favourable to continuance in evil courses. It is exceedingly important that the world's cultivation of the poppy should bear some fair relation to the world's real and scientific needs, but so long as there are many millions of people who would rather smoke opium than tobacco, or use it illegitimately in other ways, the task of limitation will be extremely difficult and the attempt at suppression hopeless. We must continue to peg away, but it is absurd to pretend that opium is no longer grown in China.

### FACTORY LEGISLATION.

MUCH useful criticism was directed to the new Factory Bill at the Conference of the Industrial Welfare Society which has just been held at Oxford. The main object of the measure is to consolidate existing factory legislation, but it contains a number of new provisions, some of them excellent, while others are open to definite objection, especially from the employers. Spitting in a factory is to be an offence punishable by fine, and, in view of the serious dangers of the practice, no one is likely to oppose such a provision. On the other hand, the new requirement that the tenant of a factory shall make "reasonable provision" for the "special medical supervision" (which includes medical treatment) of his workers is likely to bear hardly upon small employers. It is to be presumed that, in practice, this means periodical medical examination, but it would appear that the examiner is not given power to prohibit the sick person continuing to work. This and many other points are in need of elucidation, and it is already clear that the Bill will not pass without a good deal of amendment. One of the difficulties which it presents, in regard to such matters as medical examination, is that while it is precisely the small factories which most need watching, their proprietors are least able to face the additional expenditure necessitated by the measure. This seems to be one of the cases in which preliminary conference between legislators and traders would have saved a good deal of subsequent discussion, in and out of Parliament.

### WORK OF THE ASYLUMS BOARD.

THE most striking matter in the recently issued Report of the Metropolitan Asylums Board is Dr. Charles Glen's account of the after effects of that mysterious malady, encephalitis lethargica. Dr. Glen is the Assistant Medical Officer at Darenth Training Colony for improvable imbeciles and feeble minded, and he has had under observation six boys and girls, transferred as a last resort to Darenth, suffering from a serious deterioration in physique and conduct consequent upon an attack of "sleepy sickness." The relation between criminal conduct and encephalitis lethargica is a grave problem. Dr. Glen writes that "the disease may pass through a phase in which physical changes are the only prominent features, so that crimes may be committed for which the offender is not fully responsible, although he may appear, as regards intelligence, quite capable of exercising control over his actions." The position of scarlet fever was particularly satisfactory, the death-rate being only .02 per 1,000 persons living. Over 8,000 cases of diphtheria were received, and the Board feel that "means of combating this disease and of ameliorating its effects are a pressing need." Typhus fever is now being omitted from the annual tables of statistics, and enteric has become exceedingly rare. So great has been the decline in the number of cases of infectious disease that in the early summer of last year steps were taken to reduce the staffs in institutions for the treatment of these complaints and thereby materially to reduce expenditure.

### MAKING SMALLPOX EASY.

IN his recently published Annual Report, Sir George Newman, the Chief Medical Officer of the Ministry of Health, again draws attention to "the grave risks incurred in respect of smallpox as a result of the large proportion of the population which is not vaccinated." The tables which he publishes indicate quite clearly the falling percentage of vaccination to births. The Minister's Chief Medical Officer says that "the long experience of the country has taught us that beyond all question the mortality from smallpox is much less now than in pre-vaccination times; that the greatest diminution in the smallpox mortality is found in the early years of life in which there is most vaccination; and that in countries in which there is adequate vaccination and re-vaccination relatively to the population, there is little smallpox." Sir George Newman further points out that "in houses invaded by smallpox not nearly so many of the vaccinated inmates are attacked as of the unvaccinated in proportion to their numbers," and he concludes generally that though early diagnosis, prompt isolation of smallpox patients in suitable hospitals, effective disinfection, supervision of "contacts," and other such public health methods are invaluable, they are no substitute for vaccination. Yet, in face of all this, the Minister of Health has just been making easy the way of exemption from vaccination by putting into the hands of parents a form of claim to save them the trouble of applying for it. If his own Chief Medical Officer is right in saying that the protection afforded by vaccination cannot be disputed by anyone with actual experience in dealing with this disease, the Minister may reasonably be pressed for an explanation. His Order may not unfairly be said to make smallpox easy. A week or two after Mr. Wheatley's strange action, the Ministry of Health issued a statement showing that smallpox exists, or has within the last few weeks existed, in 31 places in this country. Since these places include such populous towns as Sheffield, Leeds and Leicester, and St. Pancras, the peril is obvious. It looks very much as though Mr. Wheatley and Sir George Newman were "fighting it out" behind the scenes. Yet the Minister declares that he "believes in vaccination"! It is all very puzzling.

### THE MENTALLY DEFECTIVE CHILD.

THAT the Local Education Authorities are not doing all that they might in the care and supervision of mentally defective children is common knowledge, and by no means too soon the Board of Education has been discussing the matter with the principal associations of authorities, officials, teachers and voluntary workers concerned and with other persons of special experience in the matter. The outcome is the issue of a circular by the Board containing a number of suggestions for the guidance of those who have to administer the law. Thus it is suggested that not only should authorities who have not already done so perfect their arrangements for the ascertainment of all mentally defective children in their area, but that children in attendance at special schools should be passed periodically in review in order that suitable cases may either be returned to the ordinary school or notified to the Mental Deficiency Committee. It is pointed out that when schools are not available there ought to be visitatorial supervision at home. It is lamentable that when so many mentally defective children leave school at fourteen they are under no form of public control, and spend their time aimlessly at home or mischievously in the streets. Yet up to sixteen they are within the responsibility of the Local Education Authority. Special school provision is desirable in a far larger number of cases, not only because the children in those schools enjoy education, control, and training in discipline, but because there is an opportunity to transfer them to the Mental Deficiency Committee at the age of sixteen. The individual and national perils involved in the neglect of these unfortunate young citizens are so serious that we trust that the education authorities throughout the country will not fail to take to heart the very reasonable recommendations of the Board.

### THE TEETH OF THE ARMY.

THAT "an army marches upon its stomach" is now an old story; but a stomach, like the grasshopper, is a burden unless its contents are prepared by sound teeth. Now, as we learn from the first Annual Report on the health of the Army which has been issued for ten years, our soldiers either possess no teeth or very bad ones. This belated report of the Director-General of the Army Medical Services deals only with 1921, and in that year eighty-one out of every hundred soldiers inspected were in need of dental treatment. It is the exception to find a newly joined recruit who has been in the habit of cleaning his teeth. The dental standard of the Army is not unduly high, all that is required being that the recruit should possess eleven out of the twenty-two points which are regarded as dental perfection. In the years immediately following the war the Army appears to have been the refuge of a large proportion of our C3 men, and its physical standard during 1921 was very much lower than that which existed prior to the Great War. The rejections were 354 per 1,000 after the war, as compared with 205 before it, notwithstanding that, owing to the scarcity of experienced medical officers, many men were accepted who ought to have been—and, we presume, now are being—rejected. There was five per cent. of sickness in 1921, as against three per cent. in 1913; moreover, the young recruits were so destitute of stamina that they reported sick much more readily than seasoned men. Happily there was a substantial fall in forms of disability resulting from vice—the figures were, indeed, lower than at any time in the history of the British Army. It is, moreover, encouraging to learn that there is every reason to expect a speedy return of the good showing of the old Army.

### A LAND OF MASSACRE.

WHEN the Union of Soviet Socialist Republics is not killing its subjects by rope, bayonet or bullet, it is massacring them in millions by disease. As a general statement this has long been a familiar fact, but Dr. Horsley Gantt, formerly Chief Medical Officer of the Petrograd United American Relief Administration, has provided us with some appalling details. Between 1920 and 1922 he personally saw cases of cannibalism, and quite frequently dogs and men fought in the streets of Petrograd over the carcase of a horse. Whereas the old normal Russian death-rate was twenty-five per thousand, in 1922 it rose to eighty-five. Between twenty and thirty million people had typhus fever, and three millions died of it; ten millions suffered from recurrent fever; in 1921 about 600,000 cases of typhoid, cholera and smallpox were officially registered; and the authorities themselves put the total deaths from epidemics since 1916 at between eight and ten millions. Dr. Gantt avers that, judged by Western standards, about one-half of the population of Russia require hospital treatment. Of nearly two thousand students medically examined in Kieff in the present year only fifteen per cent. were in good health. The ten thousand hospitals which existed under the old *régime* have terribly deteriorated. Though they now have more food and linen and are more orderly and cleanly, they are as badly off as ever in equipment and the number of *personnel*. Yet in face of these official figures, apologists for the bandits and cut-throats who form the present government of Russia ask the world to believe that the Russian revolution has been no worse than any of its predecessors!

### PAYING PATIENTS IN AMERICA.

IN America, as in England, the question of the paying patient in hospitals is forcing itself to the front. Opinion is, however, a good deal divided on the subject. The superintendent of a hospital in Chicago suggests that "sympathy for the people of moderate means is being somewhat overworked," on the ground not only that they are now in receipt of greatly enhanced incomes, but that when they go into hospital they make exaggerated demands. He declares that men of "moderate means"—which appears to mean artisans earning from four to nine shillings an hour—plead that they cannot afford to pay from sixteen to twenty-four shillings a day for treatment in a private room, yet turn up their noses at treatment in the cheaper quarters. Upon such persons we need not waste much sympathy, and we may be thankful that we rarely have to deal with their like in England. On the other hand, the Cornell Pay Clinic, in New York, which aims at supplying care for persons of moderate means, finds that the problem affects the greater part of the population. Consequently, although there can be no one solution of so large a difficulty, it is regarded as proved that there must be a much more extensive use of hospitals by those classes. The statement on the subject issued by the Cornell Clinic closes with the sensible remark that the public must come to look upon hospitals, not as "charities," but as institutions for the practice of medicine, not displacing bedside care in the home, "but providing all the people and all qualified physicians with facilities for better diagnosis, treatment and prevention of disease, which will render medical practice more efficient and more capable of reaching every one of those who need its benefits."

### CLEANLINESS IN SCHOOLS.

ACCORDING to Sir William Hamer there is a marked awakening among London school-children of the sense of personal hygiene, and as a natural consequence an improvement in their health. This awakening reflects great credit upon the parents and also upon the teachers, whose influence in these directions upon children under their charge may be very potent. But instruction on the laws of health to be really effective must be given in surroundings which illustrate the practical carrying out of these laws. It is useless to teach children to keep themselves clean while the buildings in which they sit are antiquated and unhygienic, and where there is a lack of proper facilities for washing. The County M.O.H. for Lancashire is only one of many medical officers demanding more cleanliness in schoolrooms. " The interior and surroundings of a school," he writes, " should be kept as clean and tidy as a private house ; the floors should be scrubbed and washed at very frequent intervals and the furnishings thoroughly dusted." Certainly many schools fall short of these requirements. The position as regards the medical inspection of school children is itself distinctly encouraging, and in the Metropolis boys and girls needing attention now receive it immediately. Doctors and school nurses are able to accomplish between them very great reforms, but it is essential for them to have the support of the school teachers, whose part it is, by precept and practice, to prevent the disease which doctor and nurse are called upon to cure.

### HEALTH TALKS AT A COUNTY FAIR.

IN the United States everyone is compelled to be interested in the public health; there is no escaping the knowledge of its laws. Even at the County Fair an enterprising public health nurse may be found in active competition with more frivolous showmen. For this work she must be a fluent talker and a person of much self-possession, and must have something to show as well as something to say. She may give some kind of demonstration such as the proper method of making a bed or bathing a baby, or she may wish to exhibit unsatisfactory conditions in the hope of stimulating a desire for improvement. Such an exhibit was used by the New York League of Women Voters to focus attention upon rural school conditions and was named " The Little Red School House Theatre." This miniature school house is set up in a tent or booth, and the first scene shows an old-fashioned and neglected school ; the second, a remodelled building. The reasons for change are explained and an effort made to enlist the support of the audience for better school conditions. Another exhibit of a somewhat similar kind displays a painted street scene, showing the work of the child hygiene nurse. In another a little train puffs along over " Healthland," up " Potato Hills " and through the county of " Oatmeal." There is, indeed, no lack of original exhibits for the nurse who is bold enough to go to the County Fair. There are still fairs in various parts of England where American methods might to some extent be adopted, or perhaps market-day might be a likely opportunity.

### BEAVER CONEY.

SKIN trouble found to be due to the wearing of certain cheap furs has been the subject of a special investigation by the Ministry of Health. The nocuous furs were of a cheap variety, made from rabbit skins which are dyed to resemble coney and are known as " beaver-coney." These furs originate abroad, and are chiefly imported from Belgium, France, Germany, and the United States. They are sent over dyed and ready for use. The sufferers from this special form of skin trouble have been exclusively women and girls varying in age from 15 to 50 and for the most part belonging to the class of patients that seeks,and finds,treatment in hospitals. Though not in most cases of serious import, the symptoms in some cases were severe and the rash, at least, disfiguring and uncomfortable. In one case reported from Leicester, the woman had as many as four attacks before the real cause was suspected. On the occasion of one attack the irritation was so intense that her husband says she tore her hair and crying out as if in agony. She herself described it as if she was being scalded. There was great œdema of the face ; indeed, for two days the eyes were completely closed. Medical attendance had to be sought and the attack lasted for twelve days, during most of which time she was in bed. It is concluded that the trouble is attributable to insufficient care having been taken in certain cases to secure that complete oxidisation of the dyeing materials was secured at the end of the process. The report is published in the hope that, when taken into account by the dyers, importers and other traders concerned, it will strengthen and make more effective the measures which should be taken within the industry to prevent the continuance of this risk.

### THE VALUE OF HOME NURSING.

IN his report for the year 1923, the Medical Officer of Health for Bristol gives pride of place to the value of Home Nursing. The most encouraging feature of the year, he says, was the success attending the work of the trained nurses employed either in the Infant Welfare and Maternity Department, or in the work of home-enquiry into the causation of disease which is now entrusted to three " Home Nurses." It is day by day becoming more evident that the most fruitful results of public health endeavour are to be gained by carrying instruction into the homes, and this can best be entrusted to trained nurses. The three nurses in Bristol visited 7,446 cases of infectious disease among children in the course of the year ; this total does not include visits subsequently paid to see that isolation of patients was being carried out, or for the supervision and treatment of contacts. In cases of diphtheria, encephalitis lethargica, cerebro-spinal fever, polio-encephalitis and anterior poliomyelitis, home contacts are placed under supervision and spray treatment by the home nurse. The value of the nurses is especially emphasised in the case of measles, and the methods employed are very thorough. When a " first case " of measles is notified from certain infant departments of the Council Schools, the whole of the class contacts are excluded from attendance at school from the ninth to the fifteenth day after onset of " first case," and placed under the supervision of the home nurse.

